# Multifaceted Aspects of HIV-1 Nucleocapsid Inhibition by TAR-Targeting Peptidyl-Anthraquinones Bearing Terminal Aromatic Moieties

**DOI:** 10.3390/v14102133

**Published:** 2022-09-27

**Authors:** Alice Sosic, Francesco Frecentese, Giulia Olivato, Daniele Rollo, Caterina Carraro, Elia Gamba, Vincenzo Santagada, Barbara Gatto

**Affiliations:** 1 Department of Pharmaceutical and Pharmacological Sciences, University of Padova, Via Francesco Marzolo 5, 35131 Padova, Italy; 2 Dipartimento di Farmacia, Università di Napoli “Federico II”, Via D. Montesano 49, 80131 Napoli, Italy

**Keywords:** 2,6-dipeptidyl-anthraquinones, TAR–RNA, HIV-1 NCp7

## Abstract

2,6-dipeptidyl-anthraquinones are polycyclic planar systems substituted at opposite ring positions by short aminoacyl side chains. Derivatives with positively charged terminal amino acids showed in vitro inhibition of HIV-1 nucleocapsid (NC) protein correlating with threading intercalation through nucleic acid substrates. We found that the variation of the terminal amino acid into an aromatic moiety has profound effects on the NC inhibition of TAR–RNA melting, granting enhanced interaction with the protein. While all compounds showed appreciable NC and TAR binding, they exhibited different strengths driven by the length of the peptidyl side chains and by the stereochemistry of the terminal tyrosine. Unexpectedly, the best inhibitors of NC-induced TAR melting, characterized by the D- configuration of tyrosine, were able to form ternary complexes without competing with TAR–NC recognition sites, as shown by native mass spectrometry experiments. Furthermore, the hydrophobicity of the terminal residue enhances membrane permeation, with positive implications for further studies on these NC–TAR-targeted compounds.

## 1. Introduction

**Figure 1:**
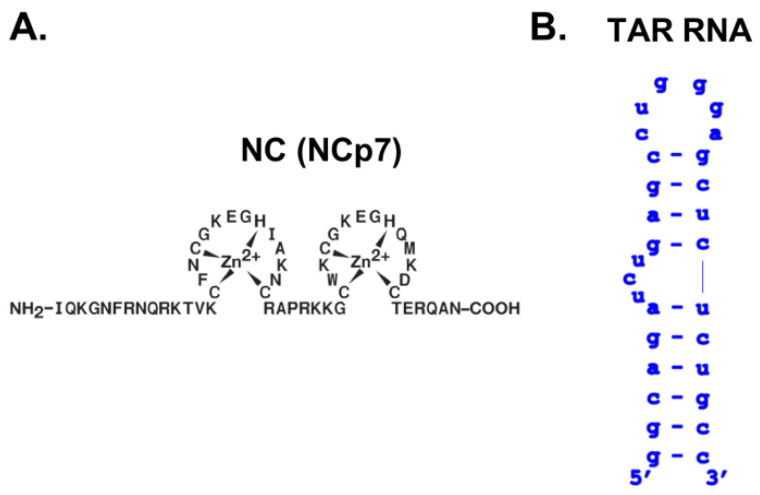
(**A**) Sequence of the full length NC protein. (**B**) Sequence and secondary structure of the TAR construct employed in this study.

The currently available anti-HIV (Human Immunodeficiency Virus) therapies exhibit clinical limitations related to the severe side effects and the emergence of resistant viral strains. For these reasons, there is an urgent need to develop novel antiretrovirals able to overcome drug resistance to optimize therapy. In this context, HIV-1 nucleocapsid NCp7 (NC), a fundamental mediator of several steps of the viral replication, demonstrated to be a valid druggable target [1]. NC is a 55 amino acids basic protein with two Zinc Fingers (ZF) (Figure 1A) and a highly flexible nature [2,3]. Due to its two highly conserved zinc-finger domains, NC exerts a *chaperone* activity over specific nucleic acids (NA) dynamic sequences, among which a relevant role is played by TAR [3,4]. TAR (trans-activation responsive element) (Figure 1B) is a stable RNA structure of the HIV-1 RNA genome, which must be paired with its complementary cTAR DNA sequence synthesized during retrotranscription. Both TAR and cTAR, folded into a hairpin structure characterized by a 3-nt bulge and a hexa-loop, are substrates of NC, which mediates their unfolding and annealing into the heteroduplex TAR/cTAR [5]. It has been shown that NC adopts a conformation in which the two ZFs are in close proximity to each other, allowing stacking between the two conserved and essential Phe16 and Trp37 of ZF1 and ZF2, respectively [6,7]. In this conformation, a small hydrophobic pocket is formed and plays a crucial role for nucleic acid chaperone properties of NC as it drives the specific interaction with unpaired guanine bases of its targets [8,9]. Belfetmi et al. analyzed in detail the interaction pattern between NC and its TAR RNA substrate: it was evidenced that the TAR regions preferentially destabilized by NC are adjacent to binding sites of low affinity for the protein, a trade-off for rapid NC on/off kinetics [10]. The UCU bulge of TAR (Figure 1B) reduces the stability of the upper and lower stem adjoining regions, that, in turn, represent suitable sites of NC-induced melting. The UCU bulge and the apical loop of TAR therefore represent moderate-to-low affinity binding sites for NC that initially interacts with the first guanine of the upper stem (position 26 as defined in [10], corresponding to G-10 in our shorter construct) made accessible by the breathing of the G-C base pair bordering the TAR bulge [10]. We thus conjectured that strong TAR binding by competing molecules could interfere with NC recognition and chaperoning.

NC thus represents a particularly attractive target to develop new classes of anti-HIV agents, as it is highly conserved and plays a central role in virus replication. Several strategies have been proposed to develop NC inhibitors [1,11]. Our main interest focused on the design of molecules that bind the nucleic acid partners of NC thereby interfering with its chaperone functions [12,13,14,15,16,17,18]. Our studies exploited threading intercalators, i.e., polycyclic ring systems able to thread through partially destabilized double stranded helices and substituted with appropriately placed side chains [19,20]. Our most active leads were 2,6-dipeptidyl-anthraquinones (2,6-AQs), whose chemical structure is characterized by an anthraquinone (AQ) nucleus bearing at opposite positions of the ring system two dipeptidyl side chains, each composed of a linker amino acid and an anchor residue constituted by a positively charged aliphatic amino acid ensuring a strong electrostatic contribution to nucleic acids binding. This class of compounds was shown to act as NC inhibitors mainly through binding and stabilization of both TAR and cTAR [12,16,21], with compounds 2,6-AQ-D-Ala-Lys/Orn that displayed not only a slightly greater affinity for the RNA rather than for the DNA substrate, but also a greater binding affinity for the bulge-loop TAR structure than for other non-related RNAs, highlighting their preferential binding to dynamic bulged regions of RNA structures [16]. Interestingly, selected lysyl-peptidyl-anthraquinones, shown by RNA footprinting analysis to recognize TAR apical loop and bulge regions, also exhibited a moderate NC binding and competition with the protein-TAR interaction, indicating other modes of NC inhibition by these threading intercalators [15]. Moreover, the early observation that an AQ bearing a β-Ala linker coupled to a *p*-aminophenylalanine aromatic anchor was a moderately active NC inhibitor while displaying a distinct profile of the inhibition curve [12], further hinted to other potential mechanisms of NC inhibition. As stacking interactions between exposed guanines and the aromatic amino acids located at the second position of the two zinc fingers (Phe16 and Trp37) have been suggested as a major driving force for NC–nucleic acid interaction [22], we speculated that derivatives with terminal aromatic anchor might be able to inhibit NC by stacking with one of the hydrophobic amino acids of the protein ZFs, thus competing with the binding to exposed bases in the nucleic acids structure [12].

**Figure 2:**
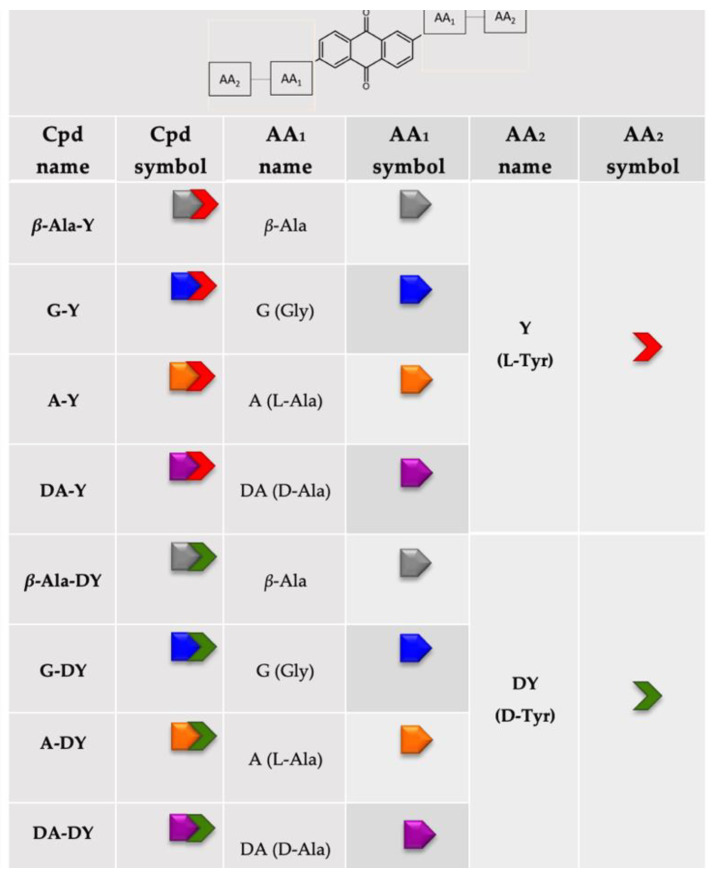
Schematic structures of the l-Tyr (Y) and d-Tyr (DY) series of 2,6-dipeptidyl−anthraquinone conjugates included in the study. The different amino acids constituting the peptidyl side chains (AA_1_ and AA_2_) are indicated by specific symbols and colours.

To verify this assumption, we designed and analyzed the eight derivatives shown in Figure 2, characterized by the formerly employed β-Ala, Gly, L-Ala and D-Ala as linker amino acid (AA_1_), and possessing the terminal Tyr of the L- or D- series as aromatic “anchor” (AA_2_). The different compounds’ side chains were coded by the specific pictograms schematized in Figure 2 and reported for clarity in all relevant figures in the text. Our experimental procedures employed fluorescence quenching assays and native mass spectrometry to assess the extent of NC inhibition, interaction with TAR, direct binding to NC protein and competition for the TAR–NC-binding sites by the l-Tyr and d-Tyr series of peptidyl-anthraquinones. Finally, we assessed whether the higher hydrophobicity of aromatic compounds would lead to a higher permeation through membranes relative to what was exhibited by the highly charged aliphatic series.

Our results highlight the importance of the terminal Tyrosine and of its stereochemistry in determining TAR recognition, NC binding and NC inhibition. The information obtained for compounds with aromatic terminal anchors is particularly interesting for the possible development of peptidyl conjugates with multiple modes of actions toward nucleocapsid protein, having positive implications for future in vivo studies.

## 2. Materials and Methods

### 2.1. Nucleic Acid Substrates and Protein

TAR is the 29-mer RNA sequence 5′-GGCAGAUCUGAGCCUGGGAGCUCUCUGCC-3′, and cTAR is its DNA complementary sequence 5′-GGCAGAGAGCTCCCAGGCTCAGATCTGCC-3′. All oligonucleotides were synthesized by Metabion International AG (Martinsried, Germany). Stock solutions were stored at −20 °C in 10 mM Tris-HCl, pH 7.5. Dilutions were made in DEPC-treated water (Ambion). When specified, oligonucleotides were labeled at 5′- and 3′-ends, respectively, by the fluorophore 5-carboxyfluorescein (FAM) and the dark quencher 4-(4′-dimethylaminophenylazo)benzoic acid (Dabcyl). Typical folding procedure consisted in the snap-cooling: TAR and cTAR diluted in the proper aqueous buffer were heated to 95 °C for 5 min and then ice-cooled in order to assume the proper hairpin structure.

The full-length recombinant NC protein was obtained as reported [23]. The protein concentration was determined on a UV−vis Nanodrop 1000 (Thermo Scientific, Waltham, MA, USA), using an extinction coefficient at 280 nm of 6410 M^−1^·cm^−1^.

### 2.2. Synthesis of 2,6-Disubstituted-Anthraquinone Derivatives

The l-Tyr and d-Tyr series of dipeptidyl-anthraquinones have been obtained by standard coupling of β-Ala, Gly, L-Ala, D-Ala, with either Fmoc-Tyr(Tbu)-OH or Fmoc-d-Tyr(Tbu)-OH via HBTU/DMAP as previously reported in literature [12,16,21]. The synthetic strategy is summarized in Scheme S1 (see Supplementary Information). The protecting groups were removed by reaction with 33% diethylamine in THF and with trifluoroacetic acid in H_2_O (9:1; *v*/*v*) giving the desired compounds that were purified by preparative RP-HPLC and were characterized by mass spectrometry. Reactions were stirred at 400 rpm by Heidolph MR Hei-Standard magnetic stirrer. Solutions were concentrated with a Buchi R-114 rotary evaporator at low pressure. RP-HPLC preparative purification was routinely performed on a Shimadzu prominence LC-20 AP system equipped with a multiwavelength prominence SPD-20A UV-VIS detector on a Supelco Ascentis^®^ C18 column (10 µm, 25cm × 21.2 mm) employing the following solvents: A: 100% acetonitrile in 0.1% TFA, B: 100% H_2_O in 0.1% TFA. The operational conditions were as follows: linear gradient of 5–70% acetonitrile + 0.1% TFA in 30 min, using a flow rate of 30 mL/min. Purity of the products was assessed by analytical RP-HPLC using a Macherey-Nagel Nucleosil 100-5 C18 column (5 μm, 4 × 125 mm). The column was connected to a Rheodyne model 7725 injector, a Shimadzu-10 ADsp HPLC system, a Shimadzu SPD-20 A/SPD-20 AV UV-VIS detector set to 254 nm. Mass spectra of the final products were performed with an LTQ Orbitrap XL™ Fourier transform mass spectrometer (FTMS) equipped with an ESI ION MAX™ source (Thermo Fisher, San José, CA, USA).

### 2.3. NC-Mediated TAR Melting

A FRET-based screening was performed to identify inhibitors of NC melting on the TAR structures shown in Figure 1B, which bore 5′-FAM and 3′-DAB modifications at each end as described in the former HTS format [12,14,15,16,21]. We used a VictorIII (PerkinElmer) microplate reader with 485 and 535 nm as excitation and emission wavelengths. 1 μM aliquot of TAR hairpin was folded by snap cooling as described above in TNMg (Tris-HCl 10 mM, NaCl 20 mM, Mg(ClO_4_)_2_ 1 mM pH 7.5). The samples were then diluted to 0.1 μM in TN (Tris-HCl 10 mM, NaCl 20 mM pH 7.5). Increasing concentrations of compound (0, 0.1, 0.5, 1, 5, 10, 50 and 100 μM final) were added to each sample before introducing NC to a final concentration of 0.8 μM (for a 1:8 oligo to NC molar ratio). The better relative ratio nucleic acid:NC resulted in being 1:8 to have the higher TAR-melting activity avoiding aggregation of oligonucleotides, in agreement with what indicated in literature [12,22,24]. The plate was immediately read at room temperature three times with 1 min intervals, unless differently specified. The experimental data were fitted as reported earlier to enable calculation of the respective IC_50_ value [21]. Each experiment was performed in triplicate to calculate an average and standard deviation. The dose–response curves resulting from the NC-mediated melting in the presence of each 2,6-AQ on TAR substrate are reported in Figure S1 (see Supporting Information).

### 2.4. ESI-MS Analysis of the Direct Binding of 2,6-Disubstituted-Anthraquinones to TAR

After a desalting step performed by using 3K NMWL (Millipore Corporation, Burlington, MA, USA) centrifugal filters to minimize the presence of salts that can adversely interfere with ESI performance, TAR was folded in 150 mM ammonium acetate (pH 7.5) as described above. Samples for binding studies were prepared by mixing appropriate volumes of folded TAR (1 μM final) with each compound in 150 mM ammonium acetate (pH 7.5). The final mixtures contained a 10:1 of 2,6 dipeptidyl- anthraquinone:substrate molar ratio. To establish the binding equilibrium in solution, samples were incubated for 15 min at room temperature before the analysis. Control experiments were performed on 1 μM solutions of TAR in 150 mM ammonium acetate. All samples were analyzed in negative ion mode by direct infusion electrospray ionization (ESI) on a Thermo Fisher Scientific (West Palm Beach, CA, USA) LTQ-Orbitrap Velos mass spectrometer. The analyses were performed in nanoflow ESI mode by using quartz emitters produced in-house by a Sutter Instruments Co. (Novato, CA, USA) P2000 laser pipet puller. Up to 6 μL samples were loaded onto each emitter by using a gel loader pipet tip. A stainless steel wire was inserted in the back- end of the emitter and used to supply an ionizing voltage ranged around 0.8−1.0 kV. Source temperature and desolvation conditions were adjusted to decrease the incidence of salt adducts, with typical source temperature of 200 °C. Data were processed by using Xcalibur 2.1 software (Thermo Scientific). The determination of free and bound RNA, necessary to evaluate the binding affinity of compounds to TAR, was assessed from the relative abundances, expressed as percentage and compared [16,25].

### 2.5. Fluorescence Quenching Assay (FQA)

The ability of the tested compounds to stabilize TAR RNA structure was measured by the increase in melting temperature of the oligoribonucleotide in the presence of different concentrations of each compound. Melting temperature (T_m_) is the temperature at which 50% molecules of oligoribonucleotide are denatured. TAR RNA hairpin was formed by snap cooling at 10 μM in TNMg (Tris-HCl 10 mM, NaCl 20 mM, Mg(ClO_4_)_2_ 1 mM pH 7.5) and then diluted to 1 μM concentration in ETN (EDTA 1 mM, Tris HCl 10 mM, NaCl 20 mM, pH 7.5). In each well, the nucleic acid solutions were mixed with the anthraquinones solutions to the final concentrations of compound 10 μM. Nucleic acid solutions without compound were used to measure the reference T_m_ value. The melting protocol consisted of a melting phase, in which the temperature increased from 25 to 99 °C in 1 h (0.02 °C/s). Fluorescence emission of FAM was read by using a Light Cycler 480 II (Roche) with emission at λ = 510 nm and correlated to the melting temperature of the oligoribonucleotide. The T_m_ value was mathematically derived from the thermal denaturing profile by using LC480 software. ΔT_m_ was calculated by using the following equation: ΔT_m_ = T_m2_ − T_m1_, where T_m2_ and T_m1_ are the T_m_ values measured by testing the RNA structure in the presence or absence of compound, respectively. Analogously, experiments were performed with folded cTAR and annealed TAR/cTAR formed upon thermal denaturation/cooling [12].

### 2.6. ESI-MS Analysis of the Direct Binding of 2,6-Disubstituted-Anthraquinones to NC

Samples for the binding studies were prepared by mixing appropriate volumes of stock solution of the full-length NC (10 μM final) with each compound in 150 mM ammonium acetate (pH 7.5). The final mixtures contained a 10:1 compound/NC molar ratio, which allowed the detection of anthraquinones binding to the protein. To ensure the binding equilibrium in solution before analysis, the samples were incubated for 15 min at room temperature. We performed control experiments using a solution of NC protein in 150 mM ammonium. All samples were analyzed in positive ion mode by direct infusion electrospray ionization (ESI) on a Synapt G2 HDMS traveling-wave ion mobility spectrometry (IMS) mass spectrometer (Waters, Manchester, UK). Mass Lynx (v 4.1, SCN781) software was used to process data. To evaluate the binding affinity of compounds to the full-length NC, free and complexed protein abundances in each experiment were calculated as reported earlier for TAR, and were finally expressed as percentage and compared. Analogously, experiments were performed with Tat protein replicating the minimal amino acid sequence (48−57) necessary for TAR binding [15].

### 2.7. ESI-MS Analysis of Ligand-RNA-Protein Ternary Complexes

Possible effects induced by 2,6-dipeptidyl anthraquinones on the specific binding of NC protein to TAR substrate were evaluated by analyzing samples in which preformed ligand–TAR complexes were challenged by addition of the remaining component. Samples were prepared by incubating equimolar amounts of NC and TAR (i.e., 10 μM concentration of each) and a 1:10 molar ratio of each ligand (i.e., final 100 μM concentration) in 150 mM ammonium acetate (pH 7.5). ESI- MS performed under nondenaturing conditions was applied to unambiguously identify all species present at equilibrium in solution. In order to characterize all the reaction products, all samples were analyzed in both positive and negative ion modes via direct infusion nanospray ionization on a Synapt G2 HDMS traveling-wave ion mobility spectrometry (IMS) mass spectrometer (Waters, Manchester, UK), using the same conditions used for the binding analysis of ligands to NC.

### 2.8. Parallel Artificial Membrane Permeation Assay (PAMPA)

The parallel artificial membrane permeation assay (PAMPA) was performed as suggested by Kansy et al. [26] with minor modifications [27]. Plates were purchased from Sigma-Aldrich as well as the components of the lipid mixture, which consisted of 1% l-α-phosphatidylcholine in n-dodecane. Compound dilutions prepared in PBS (pH 7.4) at a concentration of 500 μM from 10 mM DMSO stocks constituted the solution applied to donor wells. PBS containing 5% DMSO was employed as receiver buffer added to acceptor wells. After 24 h of incubation at room temperature, aliquots from the initial solution, donor, and acceptor compartments were analyzed by UV-Vis spectroscopy on a UV−vis Nanodrop 1000 (Thermo Scientific). The reported compartment distribution for test compounds considered the species detected in the donor, acceptor and membrane compartments (the last one indirectly determined as the difference between the relative amount of compound in the initial solution and in the aqueous compartments).

## 3. Results and Discussion

### 3.1. l-Tyr and d-Tyr Series of Dipeptidyl-Anthraquinones Inhibit NC-Mediated TAR Melting

To assess the ability of the disubstituted anthraquinones to impair the NC-mediated melting of TAR, we employed the TAR construct of Figure 1B properly double-labeled at each termini with fluorophore and quencher. NC-induced melting of the stem increases the distance between fluorophore and quencher and results in an increase in fluorescence. Therefore, the inhibition of the NC-mediated helix destabilization results in a quenching of fluorescence due to the proximity of the stem ends. After preliminary controls to ensure the absence of direct quenching by the compounds under investigation, their ability to inhibit TAR melting was evaluated in the presence of recombinant full-length HIV-1 NC. For comparison purposes, the same experiments were performed employing cTAR, the DNA complementary to TAR, as substrate of NC. The concentrations of compounds that induced 50% reduction of the stem-destabilizing activity of the protein (i.e., IC_50_) on TAR and cTAR are reported in Table 1 and Table S1, respectively.

The results obtained from the l-Tyr series evidenced that the compounds were medium to good inhibitors of the NC-mediated melting of TAR, in line with what was observed in a previous series [12,16]. All the members of the d-Tyr series were found capable of inhibiting NC-mediated melting, with IC_50_ values lower than 11 μM. A direct comparison between corresponding IC_50_ values observed for the analogous l-Tyr and d-Tyr revealed that the stereochemistry of the terminal Tyr-residue had a different impact depending on the structure of the linker. Considering NC inhibition of the TAR bulge-loop construct employed, the non-natural d-configuration in the terminal Tyr residue was slightly detrimental for the analogs bearing Ala (both l- and d- configuration) as linker, but it notably improved the potency of the compounds bearing β-Ala (β-Ala-DY) and Gly linker (G-DY), with G-DY being the most active compounds among all the d-Tyr and the l-Tyr series. The tested compounds generally resulted in being more active on TAR rather than on cTAR (Table S1) and this observation prompted us to further investigate the compounds’ interactions with the RNA substrate.

### 3.2. l-Tyr and d-Tyr Series of Dipeptidyl-Anthraquinones Directly Interact with TAR without Stabilizing Its Structure

**Figure 3:**
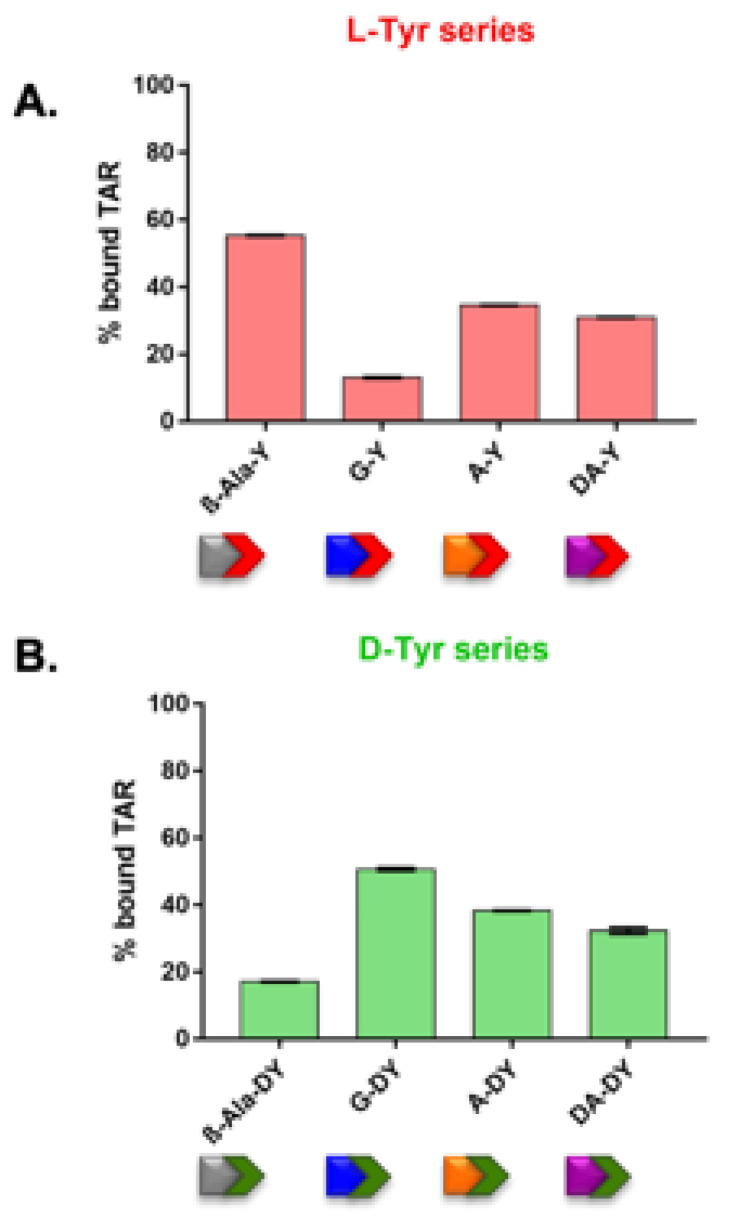
Histograms displaying the percentages of bound substrate observed in the ESI-MS spectra obtained by mixing TAR with the compounds of the l-Tyr (**A**) and the d-Tyr (**B**) series. The indicated percentages of bound substrate provide a measure of the relative affinities of l- and d-analogues for the nucleic acid substrate.

As seen in Table 1, the new series of derivatives with a terminal aromatic moiety are good inhibitors of the NC-induced melting of TAR. To verify the extent of direct RNA recognition in the new series of compounds, we evaluated their binding to TAR by employing electrospray ionization mass spectrometry (ESI-MS) under non-denaturing conditions. After preliminary controls in the absence of ligand to verify the experimental mass of the TAR construct, we analyzed sample solutions that contained 1 and 10 μM of respectively nucleic acid substrate and 2,6-dipeptidyl anthraquinone ligand (i.e., a 1:10 molar ratio). Representative ESI-MS spectra obtained from mixtures of each compound and TAR are provided in Figure S2. The results showed that all compounds bearing either l-Tyr or d-Tyr as terminal amino acid were able to bind TAR RNA displaying different stoichiometries, reported in Table S2, which revealed the presence of multiple binding sites sharing comparable affinities [16,21]. We then proceeded to calculate the relative binding affinities of the various compounds for TAR, employing the percentage of bound substrate observed in each spectrum [16,21]. The representative histograms in Figure 3 revealed that (i) all compounds exhibited an appreciable binding affinity for TAR, (ii) the stereochemistry of the terminal amino acid affecting both the binding potency and (iii) the binding stoichiometry of compound–TAR complexes (Table S2). When AA_1_ was a β-Ala, the l-configuration was beneficial, as seen in compound β-Ala-Y which showed the highest binding affinity for TAR among all the tested compounds. Instead, compounds with the Gly linker conjugated with d-Tyr (G-DY) exhibited a much higher affinity than the l-Tyr counterpart (G-Y). The configuration of terminal Tyr did not affect the compounds bearing either l-Ala or d-Ala linker, with the presence of d-Tyr terminal only slightly increasing TAR binding.

New data on TAR binding were in contrast with what was expected on the ground of the previously analyzed series of 2,6-dipeptidyl anthraquinones bearing aliphatic side chains, which showed a clear positive correlation between nucleic acid binding and NC inhibitory properties [12,16,21]. Here, on the contrary, the lack of correlation between TAR binding and NC-induced TAR melting inhibition is evident when comparing data from Figure 3 and Table 1, with the notable exception of compound G-DY which resulted in being a strong TAR binder and a good NC–TAR melting inhibitor. These results suggest that the aromatic moiety as terminal residue may change the mode of interaction with the substrate. Since in the aliphatic series NC inhibition was mainly due to TAR stem-loop stabilization upon RNA intercalation and electrostatic binding [16,21], we measured the ability of the new aromatic compounds to intercalate the nucleic acid by analyzing the thermal denaturation profile of the TAR construct by fluorescence quenching assay (FQA) in the absence/presence of compound [12,15,16,21]. Experiments were performed on folded TAR, as well as on folded cTAR and on the annealed TAR/cTAR heteroduplex for comparison. Table S3 summarizes the values of ΔT_m_ obtained from sample solutions that contained 1 μM of nucleic acid substrate and 10 μM ligand concentration. While no effects were detected on the TAR/cTAR hybrid, minimal effects were produced on cTAR T_m_ suggesting a slight stabilization of the DNA construct, which, however, resulted in being irrelevant as the compounds were poor inhibitors of the NC-induced melting of cTAR (Table S1). Interestingly, all new compounds induced minimal or undetectable effects on TAR construct T_m_ (Table S3), implying that the RNA binding of compounds bearing the aromatic terminal likely does not involve intercalation and helix stabilization, at variance with the previously analyzed compounds bearing aliphatic charged terminal residues [12,15,16,21], suggesting that the terminal tyrosine ring may prefer to engage in stacking interactions with exposed nucleotide bases in TAR structure.

### 3.3. l-Tyr and d-Tyr Series of Dipeptidyl-Anthraquinones Directly Interact with NC Protein

**Figure 4:**
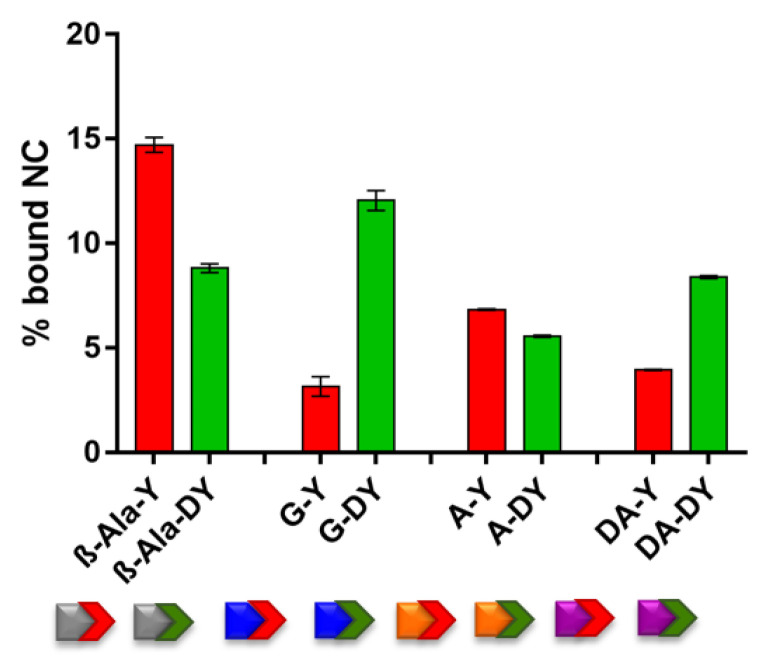
Histograms comparing the fractional occupancy of l-Tyr (red) and d-Tyr (green) series of compounds (see pictograms for side chain amino acid identities). Data were obtained from analyses of the ESI-MS spectra of reaction mixtures containing the full length NC protein and each compound (molar ratio compound: NC = 10:1) in 150 mM ammonium acetate.

To elucidate the molecular details responsible for the NC–TAR inhibition detected in vitro, we investigated the ability of AQ-l-Tyr and the AQ-d-Tyr compounds series to directly interact with the NC protein in the absence of the nucleic acid substrate. ESI-MS analyses were carried out under nondenaturing conditions to enable the detection of complexes between compounds and intact zinc-bound NC [14,16,28]. Figure 4 shows the percentage of bound NC detected in the ESI-MS spectra (Figure S3) obtained by mixing the full length NC protein with the indicated compound (molar ratio compound:NC = 10:1).

The results clearly showed that all compounds analyzed were capable of binding to the full-length NC to form stable 1:1 complexes (Figure S3). The length of the side chain combined with the stereochemistry of the terminal Tyr produced markedly different effects on the binding to NC, as the longer side chain of the β-Ala linker was especially beneficial for l-Tyr configuration, while d-Tyr yielded a medium NC binder (see β-Ala-Y and β-Ala-DY in Figure 4). For the other compounds in Figure 4 bearing shorter side chains, the absolute configuration of the terminal Tyr plays a particularly relevant effect in the case of Gly-bearing compounds: d- configuration increased dramatically the binding affinity of G-DY to NC compared to its l-Tyr counterpart GY. The binding to NC of Ala-containing compounds was instead less affected and always lower, with only DA-DY exhibiting medium NC recognition.

In addition, to evaluate the specificity to NC, we analyzed the binding of the tested anthraquinones to another HIV-1 protein capable of binding TAR, i.e., the HIV-1 trans-activator of transcription (Tat) factor. This small and positively charged protein is crucial for the efficient transcription of the integrated proviral genome [29,30,31,32], as well as to support the chaperoning activities of NC during reverse transcription [33,34]. The experiments were carried out mixing Tat with each compound, and then analyzing the samples by ESI-MS under nondenaturing conditions. The results showed unambiguously that the test ligands were not capable of binding directly to Tat (Figure S4 and Table S4), differently from what was observed for the NC protein.

Interestingly, the averaged extent of the direct interaction with the NC protein exhibited by these newly analyzed compounds resulted in being quite enhanced compared to those of the aliphatic series of 2,6-dipeptidyl anthraquinones [16], confirming that the introduction of an aromatic terminal residue is beneficial to directly targeting NC. Nevertheless, the correlation between NC binding and NC inhibition (Figure 4 and Table 1) among the l- and d-Tyr series is not straightforward, suggesting again that multiple molecular mechanisms of actions may account for the observed NC inhibitory activity.

### 3.4. Multifaceted Aspects of NC Inhibition by l-Tyr and d-Tyr Series of Dipeptidyl-Anthraquinones

**Figure 5:**
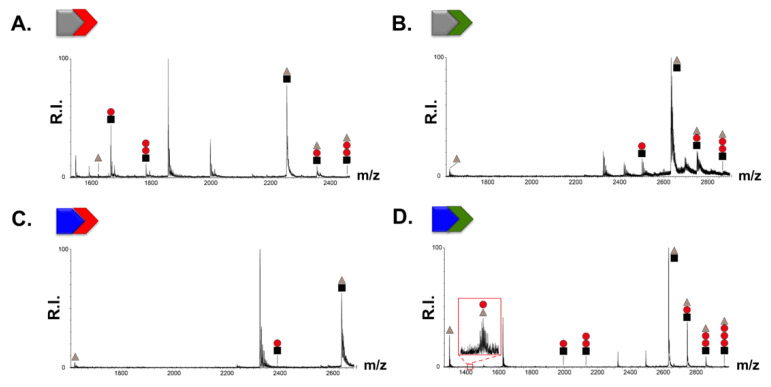
ESI-MS spectra of samples obtained by adding NC to the preformed ligand•TAR complexes. The tested compounds are (**A**) β-Ala-Y, (**B**) β-Ala-DY, (**C**) G-Y and (**D**) G-DY. Lower intensity signals near free/bound species consist of typical sodium and ammonium adducts. Grey ▲ corresponds to NC protein; black ■ to the TAR RNA substrate; and red • to the ligand. For the sake of clarity, only the 5+ and 6+ charge states of the binary (RNA-ligand) and ternary (RNA-ligand–protein) complexes are labeled in the spectra.

Results from Figure 3 and Figure 4 revealed that the compounds of the l-Tyr and the d-Tyr series can directly interact with TAR and with NC, and that the absolute configuration of the terminal aromatic moiety plays an important role. However, even if it is possible to observe an averaged binding affinity of the compounds higher for TAR rather than for NC, the potencies of the binary molecular interactions do not satisfactorily explain the effectiveness of NC-induced melting inhibition of TAR by the different compounds (Table 1). NC inhibition was particularly remarkable for G-DY, a compound showing both good TAR and good NC binding; G-Y was a weak binder of both TAR and NC, and, consistently, a bad NC inhibitor. Following this reasoning, we would have expected β-Ala-Y, also exhibiting very good TAR binding and good NC recognition, to be an efficient NC inhibitor, which was not the case as this compound was instead the weakest NC inhibitor of the whole series. To further complicate the scenario, β-Ala-DY, a bad TAR binder and a medium NC binder, was quite a good NC inhibitor. To shed light on these contradicting results, we employed competition experiments to evaluate how preformed compound–TAR complexes were challenged by NC protein addition. The outcome of each competition was determined by ESI-MS under native conditions, a method shown for enabling the simultaneous detection of non-covalent binary complexes between RNA and NC and of nucleic acid-protein–ligand ternary complexes [16]. Control experiments were performed by incubating equimolar amounts of full-length NC protein with TAR construct in the absence of compound, which confirmed the formation of stable 1:1 TAR•NC complexes [16,35]. Representative spectra obtained from a mixture of equimolar concentration of NC and ligand-pretreated TAR (10 μM) are reported in Figure 5 and Figure S5. Spectra enabled the unambiguous identification of all species present at equilibrium in solution.

Different scenarios were detected for the different compounds. Considering compounds with β-Ala- and G- as AA_1_, l-Tyr (Y) or d-Tyr (DY) as AA_2_ significantly changed the outcome of the competition experiments (Figure 5A–D). We identified the binary ligand–TAR and NC–TAR complexes as well as the ternary ligand–TAR–NC complex in all cases except G-Y, a very weak NC binder and NC inhibitor that did not display the ternary complex (Figure 5C). The stoichiometry of complexes was quite revealing: β-Ala-Y exhibited both the binary complex with TAR and the ternary complexes with NC, exhibiting in both cases the same stoichiometries (up to 2:1 ratio compound:TAR) (Figure 5A). Interestingly, these stoichiometries did not match those observed by β-Ala-Y bound to TAR in the experiments performed in the *absence* of the protein (Table S2), where we could detect up to 3:1 compound:TAR binding stoichiometries. NC, therefore, can compete with one of the three TAR binding sites of β-Ala-Y replacing the ligand from TAR in the ternary complex. Nevertheless, this site competition was not crucial to impair NC melting activity, as β-Ala-Y is one of the weakest NC–TAR melting inhibitors.

Instead considering the d-Tyr analogs β-Ala-DY (Figure 5B) and G-DY (Figure 5D), both compounds clearly displayed in their competition spectra an *additional* binding stoichiometry in the ternary complexes compared to the binary ones. As seen in the spectra 5B and 5D, β-Ala-DY and G-DY showed respectively 2:1 and 3:1 compound:TAR equivalents after NC was added to the incubation mix, while the control experiments performed in the absence of the protein showed 1:1 and 2:1 binding stoichiometries, respectively, for the TAR complexes (Table S2). This unexpected result suggests that NC loaded with the two above-mentioned compounds was able to bind TAR without competing for a ligand binding site(s), or, alternatively, that NC binding to its substrate does create a new binding site in the ternary complex that is suitable for these two compounds with d-Tyr configuration. Notably, in the case of G-DY, we could also detect the binary ligand-NC complex in the spectrum, highlighting the efficiency of G-DY in binding the protein even in the presence of TAR. The formation of ternary complexes by β-Ala-DY and G-DY correlates with data in Table 1 showing that β-Ala-DY and G-DY were the best inhibitors of the NC-induced melting of TAR, indicating the importance of the side chain length and of the absolute configuration of the terminal tyrosine amino acid for this peculiar mode of action. The four compounds bearing a methyl substituent in the linker (l-Ala or d-Ala), which were weaker TAR and NC binders, exhibited the same pattern of stoichiometry in the competition experiments regardless of the absolute configuration of the terminal Tyr (Figure S5): in addition to the binary ligand–TAR and NC–TAR complexes, ternary complexes between NC protein, TAR, and the ligand were detected, and they were the same observed in the experiments performed in the absence of the protein (Table S2), consistently with their poorer NC inhibition (Table 1).

### 3.5. Cell Permeability of d-Tyr Series of Dipeptidyl-Anthraquinones

Previous studies conducted on the series of anthraquinone derivatives bearing aliphatic doubly charged terminal residues showed a limited cell permeation and a low bioavailability in cells [12]. The introduction of the Tyr residue decreases the charge of the peptidyl side chains and increases the lipophilicity of the new molecules, with possible favorable effects on cell permeation. Therefore, we further investigated the d-Tyr series of compounds to determine their apparent membrane permeability. PAMPA, the acronym of Parallel Artificial Membrane Permeation Assays, is a well-validated in vitro model that, using an artificial lipid membrane, provides information about the ability of small molecules to exploit the passive transport through the cell membranes. Compound solutions are added to a donor well, while the artificial membrane at the bottom of the well allows permeation toward the acceptor well according to the compounds’ physicochemical properties. Ultraviolet−visible (UV−vis) analysis was employed for a fast quantification of the analyte in each compartment. Results reported in Figure S6 show the compartment distribution of the compounds after 24 h of incubation. Clearly, the compounds with the Tyr residue exhibited the ability to leave the donor compartment and permeate the membrane. While most of them were trapped in the phospholipidic layer and barely detected in the acceptor compartment, a significant amount of G-DY was detected in the acceptor compartment, suggesting its favorable permeation. On the contrary, the anthraquinone derivatives bearing aliphatic and doubly charged side chains, which were included in the PAMPA assays as reference, were not able to diffuse to the membrane, which is consistent with their poorer permeability [12].

## 4. Conclusions

The studies conducted on the compounds in Figure 2 highlight different modes of interaction of these conjugates with TAR and NC relative to the former series of compounds. All new compounds were able to bind TAR with a lower affinity than the earlier “canonical” aliphatic peptidyl-anthraquinones, and all of them showed a negligible ability to thread and stabilize TAR structure. Consistently with our initial hypothesis, they showed appreciable NC direct binding, although this was strongly modulated by the side chain structures. The strongest NC binder of the series was a competitor for at least one NC–TAR binding site, but, unexpectedly, competition did not lead to effective inhibition of TAR melting. Rather, the best inhibitors among our set were those with slightly lower NC-binding potencies yet were able to form complexes with TAR and NC *without* competing with TAR–NC binding.

Based on our results, we suggest that the dipeptidyl-anthraquinones bearing a terminal aromatic moiety exhibit multifaceted binding with the protein and the RNA, driven by the length of the peptidyl side chains and by the stereochemistry of the terminal aromatic moiety, which is mainly responsible for the interaction with NC. Clearly, as the compounds are characterized by several planar surfaces (i.e., the polycyclic ring and the two distal flat surfaces of Tyr rings), there are several possible sites of stacking with the aromatic amino acids on the two zinc fingers and/or with guanines or other exposed bases in the upper stem loop of TAR, so that different complexes could be present. It has been reported that the binding of ZF2 of NC to G bordering the upper stem of TAR may position the ZF1 toward this short stem triggering its destabilization and the ensuing TAR melting [10]. Moreover, NC does not need to bind to high-affinity sites to destabilize RNA structures [10,36]. G-DY might possess the right conformation and configuration to bind at least one of the hydrophobic amino acids of the ZFs with low affinity. It is tempting to speculate that G-DY bound to NC can be brought in the complex by the protein interacting with TAR *without* competing for the same site(s). When being placed in the complex, G-DY may be positioned in the appropriate way to interfere with NC destabilization of the upper stem through its second DY moiety or with the surface of the anthraquinone ring. β-Ala-DY can still adopt this type of *productive* conformation with NC leading to inhibition, although the longer side chain weakens the interaction of the terminal DY with TAR and its positioning in the complex, resulting in a decrease in its inhibition potency. In both cases the absolute configuration of the terminal aromatic moiety is fundamental for this peculiar mode of inhibition, as the natural (L) configuration of Y in the β-Ala conjugate fails to produce the desired inhibition effect. Our data do not indicate the details of binding mode in the binary and ternary complex and detailed biophysical studies are needed to shed light on this issue. We hope that our results may be useful to better clarify the molecular requirements for effective inhibition of NC processing of its RNA substrates.

## Figures and Tables

**Table 1:** Inhibition of NC-mediated melting of TAR by AQ-AA_1_-l-Tyr (red columns) and AQ-AA_1_-d-Tyr (green columns).

AQ-AA_1_-l-Tyr	IC_50_ TAR (μM)	AQ-AA_1_-d-Tyr	IC_50_ TAR (μM)
**β-Ala-Y**	15.7 ± 0.64	**β-Ala-DY**	7.34 ± 0.31
**G-Y**	11.8 ± 0.29	**G-DY**	5.93 ± 0.63
**A-Y**	8.59 ± 0.09	**A-DY**	10.9 ± 0.93
**DA-Y**	7.85 ± 0.44	**DA-DY**	8.60 ± 0.79

## Supplementary Materials

The following supporting information can be downloaded at: https://www.mdpi.com/article/10.3390/v14102133/s1, Scheme S1: General synthetic pathway for the preparation of the anthraquinone derivatives included in the study and their characterization. Figure S1: Dose–response curves resulting from the NC-mediated melting in the presence of 2,6-AQs on TAR substrate. Table S1: Inhibition of NC-mediated melting of cTAR by AQ-AA_1_-l-Tyr (red columns) and AQ-AA_1_-d-Tyr (green columns). Figure S2: Representative ESI-MS spectra of samples obtained by mixing TAR with compounds of the l-Tyr and d-Tyr series. Table S2: Binding stoichiometries of compound-nucleic acid complexes detected in the ESI-MS spectra. Table S3: Variations of TAR, cTAR, and TAR/cTAR hybrid melting temperature (ΔTm) in the presence of an increasing concentration of the compounds of the l-Tyr and d-Tyr series. Figure S3: Representative ESI-MS spectra of samples obtained by mixing NC with compounds of the l-Tyr and d-Tyr series. Figure S4: Representative ESI-MS spectra of samples obtained by mixing Tat with compounds of the l-Tyr and d-Tyr series. Table S4: Experimental and calculated monoisotopic masses (Da) of 2,6-AQ:Tat complexes. Figure S5: ESI-MS spectra of samples obtained by adding NC to the preformed ligand•TAR complexes. Figure S6: Parallel Artificial Membrane Permeation Assays (PAMPA).
