# Supplementary material for: Multifaceted Aspects of HIV-1 Nucleocapsid Inhibition by TAR-Targeting Peptidyl-Anthraquinones Bearing Terminal Aromatic Moieties
Source: Viruses. 2022 Sep 27;14(10):2133. doi: 10.3390/v14102133 (PMC9607300; doi:10.3390/v14102133)

Supporting Information

Multifaceted aspects of HIV-1 nucleocapsid inhibition by TAR-targeting peptidyl-anthraquinones bearing terminal aromatic moieties

Alice Sosic [1], Francesco Frecentese [2], Giulia Olivato [1], Caterina Carraro [1], Elia Gamba [1], Daniele Rollo [1] and Barbara Gatto [1,*]

[1] Department of Pharmaceutical and Pharmacological Sciences, University of Padova, Via Francesco Marzolo 5, 35131 Padova (Italy)

[2] Dipartimento di Farmacia, Università di Napoli "Federico II" Via D. Montesano 49, 80131 Napoli (Italy)
* Correspondence: barbara.gatto@unipd.it

Table of content:

Scheme S1: General synthetic pathway for the preparation of the anthraquinone derivatives included in the study and their characterization. **P.2**

Figure S1: Dose-response curves resulting from the NC-mediated melting in the presence of 2,6-AQs on TAR substrate. **P.4**

Table S1: Inhibition of NC-mediated melting of cTAR by AQ-AA_1-L-Tyr (red columns) and AQ-AA_1-D-Tyr (green columns). **P.5**

Figure S2: Representative ESI-MS spectra of samples obtained by mixing TAR with compounds of the L-Tyr and D-Tyr series. **P.6**

Table S2: Binding stoichiometries of compound-nucleic acid complexes detected in the ESI-MS spectra. **P.7**

Table S3: Variations of TAR, cTAR and TAR/cTAR hybrid melting temperature (ΔTm) in the presence of an increasing concentration of the compounds of the L-Tyr and D-Tyr series. **P.7**

Figure S3: Representative ESI-MS spectra of samples obtained by mixing NC with compounds of the L-Tyr and D-Tyr series. **P.8**

Figure S4: Representative ESI-MS spectra of samples obtained by mixing Tat with compounds of the L-Tyr and D-Tyr series. **P.9**

Table S4: Experimental and calculated monoisotopic masses (Da) of 2,6-AQ:Tat complexes. **P.10**

Figure S5: ESI-MS spectra of samples obtained by adding NC to the preformed ligand•TAR complexes. **P.11**

Figure S6: Parallel Artificial Membrane Permeation Assays (PAMPA). **P.12**

Scheme S1. General synthetic pathway for the preparation of the anthraquinone derivatives included in the study and their characterization.

Reagents and conditions: (i) HBTU, DMAP, DMF; (ii) 33% N,N-diethylamine, THF; (iii) TFA/H_2O.

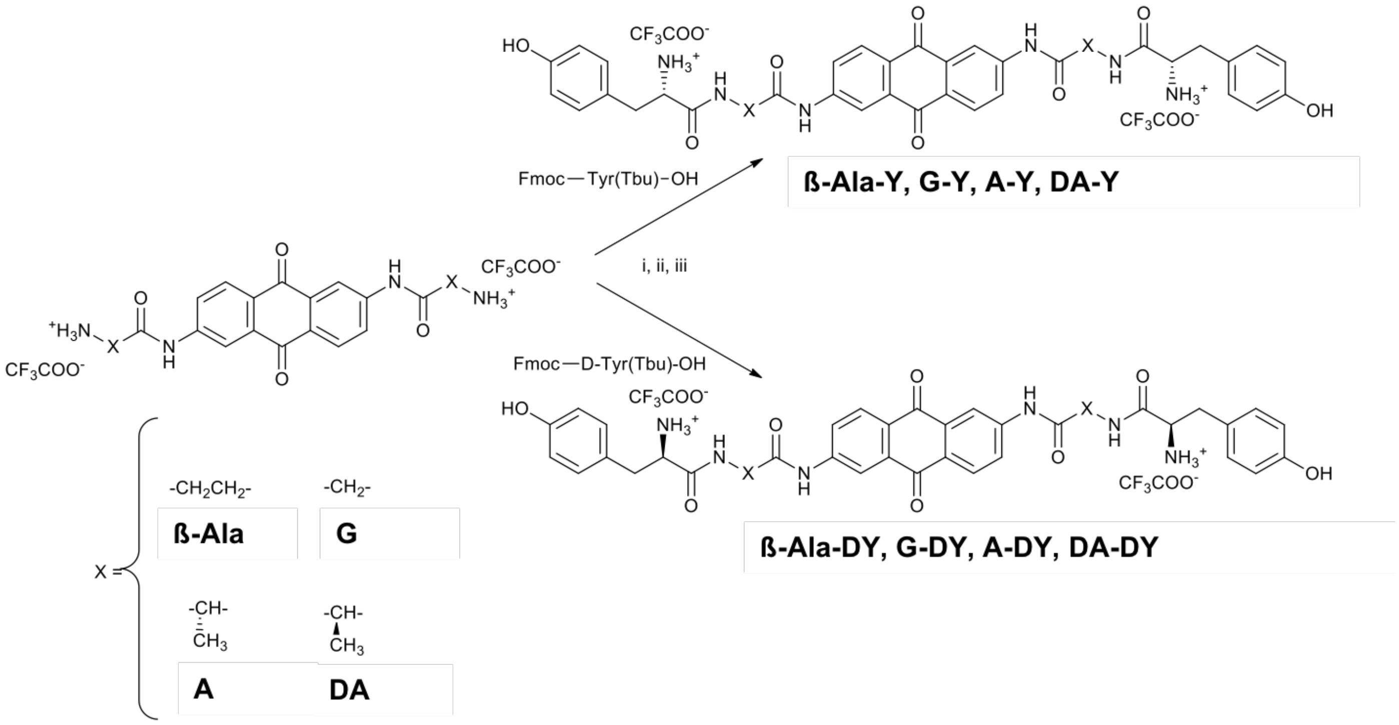

(2S,2'S)-N,N'-(((9,10-dioxo-9,10-dihydroanthracene-2,6-diyl)bis(azanediyl))bis(3-oxopropane-3,1-diyl))bis(2-amino-3-(4-hydroxyphenyl)propanamide)-bis-trifluoroacetate (ß-Ala-Y)

N,N'-(9,10-dioxo-9,10-dihydroanthracene-2,6-diyl)bis(3-aminopropanamide)-bis-trifluoroacetate (**ß-Ala**, 128 mg, 0.21 mmol) was dissolved in dry DMF (9 mL), and DMAP (282 mg, 2.31 mmol), Fmoc-Tyr(Tbu)-OH (965 mg, 2.1 mmol), and HBTU (796 mg, 2.1 mmol) were added. The resulting solution was stirred at room temperature for 24 hours, then poured into Et_2O and centrifuged. The solid obtained was washed with Et_2O and the final product was dried in vacuo and used in the next step without any further purification. In order to remove the Fmoc- protecting group, the solid obtained was reacted with a 33% diethylamine solution in THF (20 mL) for 2 hours, poured into Et_2O and centrifuged. The solid obtained was washed with Et_2O and water and then dried in vacuo. Subsequently, the obtained solid was reacted with a solution of trifluoroacetic acid in water (9:1, v/v, 10 mL) for 1 hour and then poured into Et_2O. The resulting suspension was centrifuged. The solid obtained was washed with Et_2O and dried in vacuo. Purification by preparative RP-HPLC afforded the pure compound **ß-Ala-Y** as an intense orange solid. Yield: 68%. Orange solid. K'(HPLC): 4.6. ESI-MS: 707.4 $[M + H]^+$; 354.4 $[M + 2H]^{++}$.

(2S,2'S)-N,N'-(((9,10-dioxo-9,10-dihydroanthracene-2,6-diyl)bis(azanediyl))bis(2-oxoethane-2,1-diyl))bis(2-amino-3-(4-hydroxyphenyl)propanamide)-bis-trifluoroacetate (G-Y)

The compound was obtained with the same coupling procedure adopted for **ß-Ala-Y**, starting from **G** and Fmoc-Tyr(Tbu)-OH. Yield: 64%. Orange solid. K'(HPLC): 4.7. ESI-MS: 679.4 $[M + H]^+$; 340.3 $[M + 2H]^{++}$.

(2S,2'S)-N,N'-((2S,2'S)-((9,10-dioxo-9,10-dihydroanthracene-2,6-diyl)bis-(azanediyl))bis(1-oxopropane-2,1-diyl))bis(2-amino-3-(4-hydroxyphenyl)-propanamide)-bis-trifluoroacetate (A-Y)

The compound was obtained with the same coupling procedure adopted for **ß-Ala-Y**, starting from **A** and Fmoc-Tyr(Tbu)-OH. Yield: 56%. Orange solid. K′ (HPLC): 4.3. ESI-MS: 707.4 $[M + H]^{+}$; 354.2 $[M + 2H]^{++}$.

(2S,2'S)-N,N'-((2R,2'R)-((9,10-dioxo-9,10-dihydroanthracene-2,6-diyl)bis-(azanediyl))bis(1-oxopropane-2,1-diyl))bis(2-amino-3-(4-hydroxyphenyl)-propanamide)-bis-trifluoroacetate (DA-Y)

The compound was obtained with the same coupling procedure adopted for **ß-Ala-Y**, starting from **DA** and Fmoc-Tyr(Tbu)-OH. Yield: 62%. Orange solid. K′ (HPLC): 4.4. ESI-MS: 707.6 $[M + H]^{+}$; 354.5 $[M + 2H]^{++}$.

(2R,2'R)-N,N'-(((9,10-dioxo-9,10-dihydroanthracene-2,6-diyl)bis(azanediyl))bis(3-oxopropane-3,1-diyl))bis(2-amino-3-(4-hydroxyphenyl)propanamide)-bis-trifluoroacetate (ß-Ala-DY)

The compound was obtained with the same coupling procedure adopted for **ß-Ala-Y**, starting from **ß-Ala** and Fmoc-D-Tyr(Tbu)-OH. Yield: 59%. Orange solid. K′(HPLC): 4.6. ESI-MS: 707.3 $[M + H]^{+}$; 354.3 $[M + 2H]^{++}$.

(2R,2'R)-N,N'-(((9,10-dioxo-9,10-dihydroanthracene-2,6-diyl)bis(azanediyl))bis(2-oxoethane-2,1-diyl))bis(2-amino-3-(4-hydroxyphenyl)propanamide)-bis-trifluoroacetate (G-DY)

The compound was obtained with the same coupling procedure adopted for **ß-Ala-Y**, starting from **G** and Fmoc-D-Tyr(Tbu)-OH. Yield: 55%. Orange solid. K′(HPLC): 4.6. ESI-MS: 679.3 $[M + H]^{+}$; 340.3 $[M + 2H]^{++}$.

(2R,2'R)-N,N'-((2S,2'S)-((9,10-dioxo-9,10-dihydroanthracene-2,6-diyl)bis-(azanediyl))bis(1-oxopropane-2,1-diyl))bis(2-amino-3-(4-hydroxyphenyl)-propanamide)-bis-trifluoroacetate (A-DY)

The compound was obtained with the same coupling procedure adopted for **ß-Ala-Y**, starting from **A** and Fmoc-D-Tyr(Tbu)-OH. Yield: 54%. Orange solid. K′ (HPLC): 4.3. ESI-MS: 707.3 $[M + H]^{+}$; 354.3 $[M + 2H]^{++}$.

(2R,2'R)-N,N'-((2R,2'R)-((9,10-dioxo-9,10-dihydroanthracene-2,6-diyl)bis-(azanediyl))bis(1-oxopropane-2,1-diyl))bis(2-amino-3-(4-hydroxyphenyl)-propanamide)-bis-trifluoroacetate (DA-DY)

The compound was obtained with the same coupling procedure adopted for **ß-Ala-Y**, starting from **DA** and Fmoc-D-Tyr(Tbu)-OH. Yield: 49%. Orange solid. K′ (HPLC): 4.4. ESI-MS: 707.4 $[M + H]^{+}$; 354.5 $[M + 2H]^{++}$.

Figure S1. Dose-response curves resulting from the NC-mediated melting in the presence of 2,6-AQs on TAR substrate.

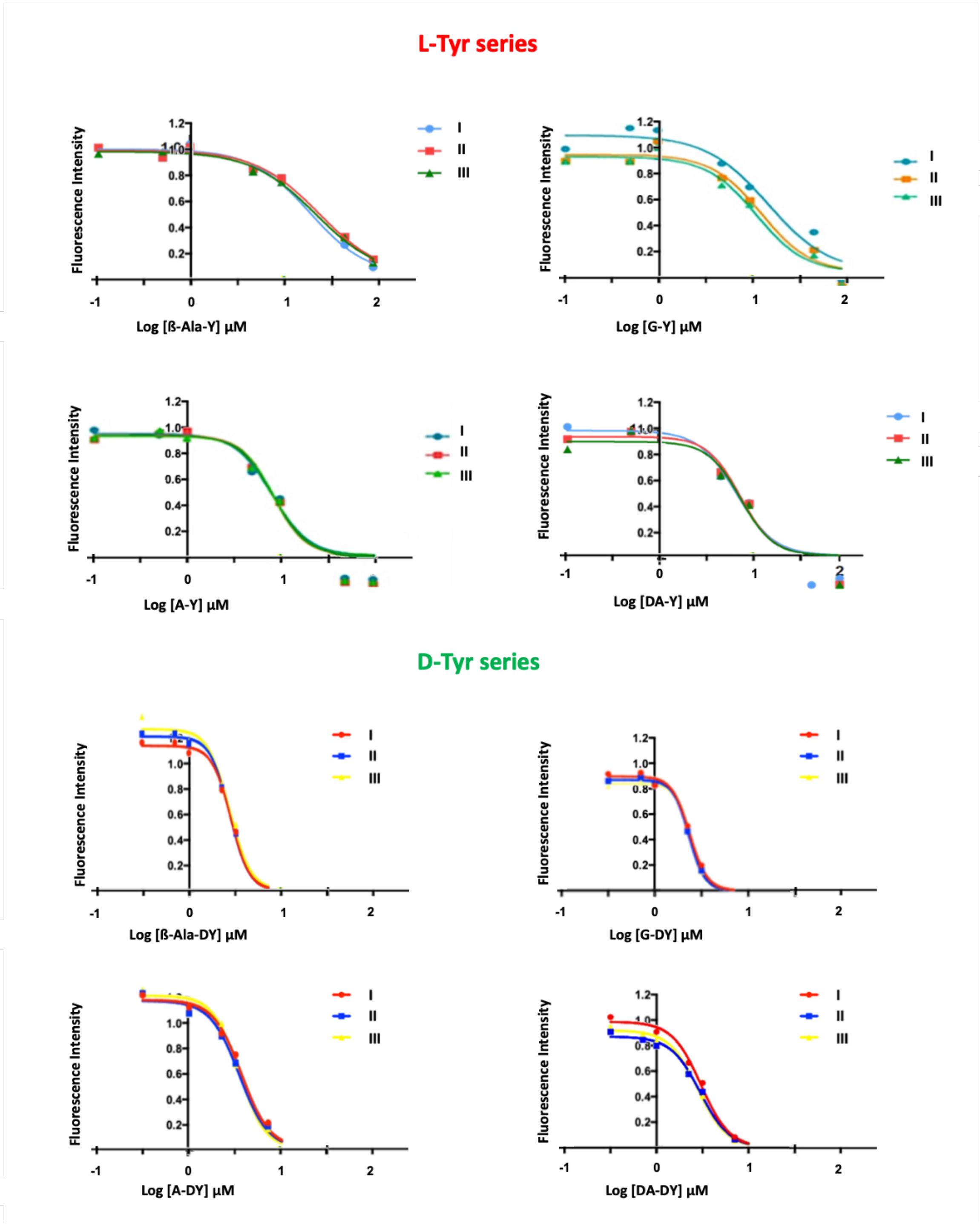

Table S1. Inhibition of NC-mediated melting of cTAR by AQ-AA$_1$-L-Tyr (red columns) and AQ-AA$_1$-D-Tyr (green columns).

AQ-AA$_1$-L-Tyr	IC_{50} cTAR (µM)	AQ-AA$_1$-D-Tyr	IC_{50} cTAR (µM)
**ß-Ala-Y**	19.4 ± 0.40	**ß-Ala-DY**	8.17 ± 0.53
**G-Y**	15.2 ± 1.49	**G-DY**	5.14 ± 0.28
**A-Y**	10.7 ± 0.31	**A-DY**	10.4 ± 1.73
**DA-Y**	8.85 ± 0.51	**DA-DY**	10.2 ± 0.86

Figure S2. Representative ESI-MS spectra of samples obtained by mixing TAR with compounds of the L-Tyr and D-Tyr series. In the figure are reported the ESI-MS spectra acquired in negative ion mode of samples obtained by mixing 1 µM of TAR with 10 µM of (**A**) **ß-Ala-Y**, (**B**) **G-Y**, (**C**) **A-Y**, (**D**) **DA-Y**, (**E**) **ß-Ala-DY**, (**F**) **G-DY**, (**G**) **A-DY**, (**H**) **DA-DY** in 150 mM ammonium acetate. Symbol ■ corresponds to TAR RNA substrate and the red ● to the ligand. The stoichiometry of bound species is graphically represented in each spectrum. Lower intensity signals near free/bound species consist of typical sodium and ammonium adducts.

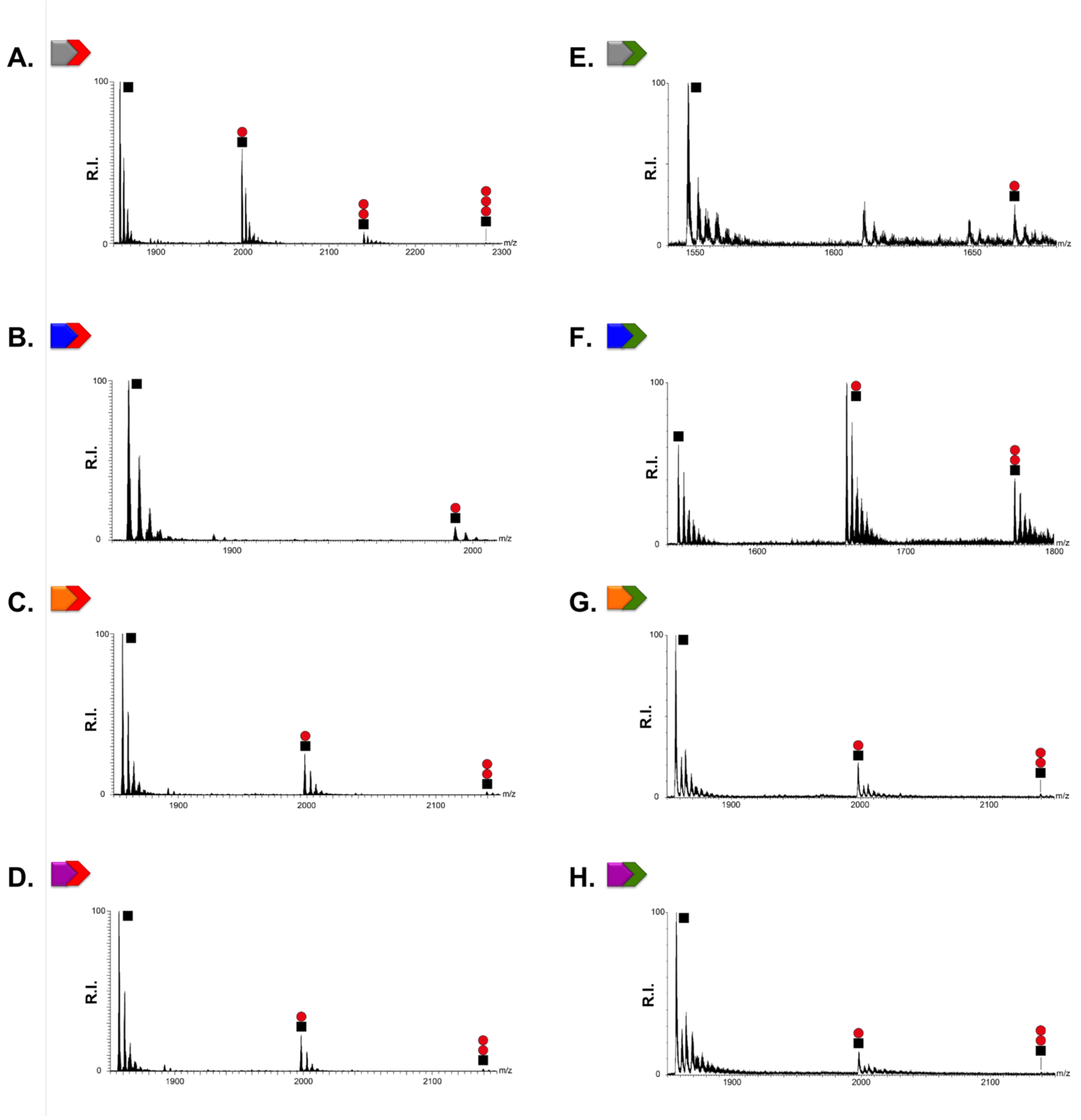

Table S2. Binding stoichiometries of compound-nucleic acid complexes detected in the ESI-MS spectra. The interactions established by AQ-L-Tyr and AQ-D-Tyr compounds with TAR constructs employed as target produced different binding stoichiometries. Data are elaborated from the negative ion mode spectra obtained for 10:1 anthraquinone:TAR molar ratio in 150 mM ammonium acetate.

Compound	1:1 AQ:TAR	2:1 AQ:TAR	3:1 AQ:TAR
**ß-Ala-Y**	X	X	X
**G-Y**	X		
**A-Y**	X	X	
**DA-Y**	X	X	
**ß-Ala-DY**	X		
**G-DY**	X	X	
**A-DY**	X	X	
**DA-DY**	X	X	

Table S3. Variations of TAR melting temperature (ΔT_m) in the presence of an increasing concentration of the compounds of the L-Tyr and D-Tyr series. Reported values are the mean ± standard error of the mean (SEM) of triplicate experiments performed on samples containing 1 µM of nucleic acid substrate and 10 µM of compound. The reference T_m value for TAR, cTAR and the TAR/cTAR hybrid in the absence of compound are 69.3, 53.8 and 69.4 °C, respectively.

L-Tyr	ΔT_m TAR (°C)	ΔT_m cTAR (°C)	ΔT_m TAR/cTAR (°C)	D-Tyr	ΔT_m TAR (°C)	ΔT_m cTAR (°C)	ΔT_m TAR/cTAR (°C)
**ß-Ala-Y**	0.03 ± 0.05	0.23 ± 0.27	0.60 ± 0.05	**ß-Ala-DY**	2.62 ± 0.11	5.81 ± 0.18	1.06 ± 0.14
**G-Y**	1.29 ± 0.04	2.38 ± 0.24	1.19 ± 0.28	**G-DY**	2.05 ± 0.20	3.34 ± 1.31	0.94 ± 0.04
**A-Y**	2.88 ± 0.04	4.48 ± 0.14	1.10 ± 0.24	**A-DY**	0.40 ± 0.74	2.50 ± 1.44	0.41 ± 0.06
**DA-Y**	0.52 ± 0.05	2.32 ± 0.03	1.10 ± 0.01	**DA-DY**	0.34 ± 0.09	2.37 ± 0.53	0.41 ± 0.11

Figure S3. Representative ESI-MS spectra of samples obtained by mixing NC with compounds of the L-Tyr and D-Tyr series. In the figure are reported the ESI-MS spectra acquired in positive ion mode of samples obtained by mixing 10 µM of NC with 100 µM of (**A**) **ß-Ala-Y**, (**B**) **G-Y**, (**C**) **A-Y**, (**D**) **DA-Y**, (**E**) **ß-Ala-DY**, (**F**) **G-DY**, (**G**) **A-DY**, (**H**) **DA-DY** in 150 mM ammonium acetate. The grey ▲ symbol corresponds to NC protein and the red ● to the ligand. The stoichiometry of bound species is graphically represented in each spectrum. Lower intensity signals near free/bound species consist of typical sodium and ammonium adducts.

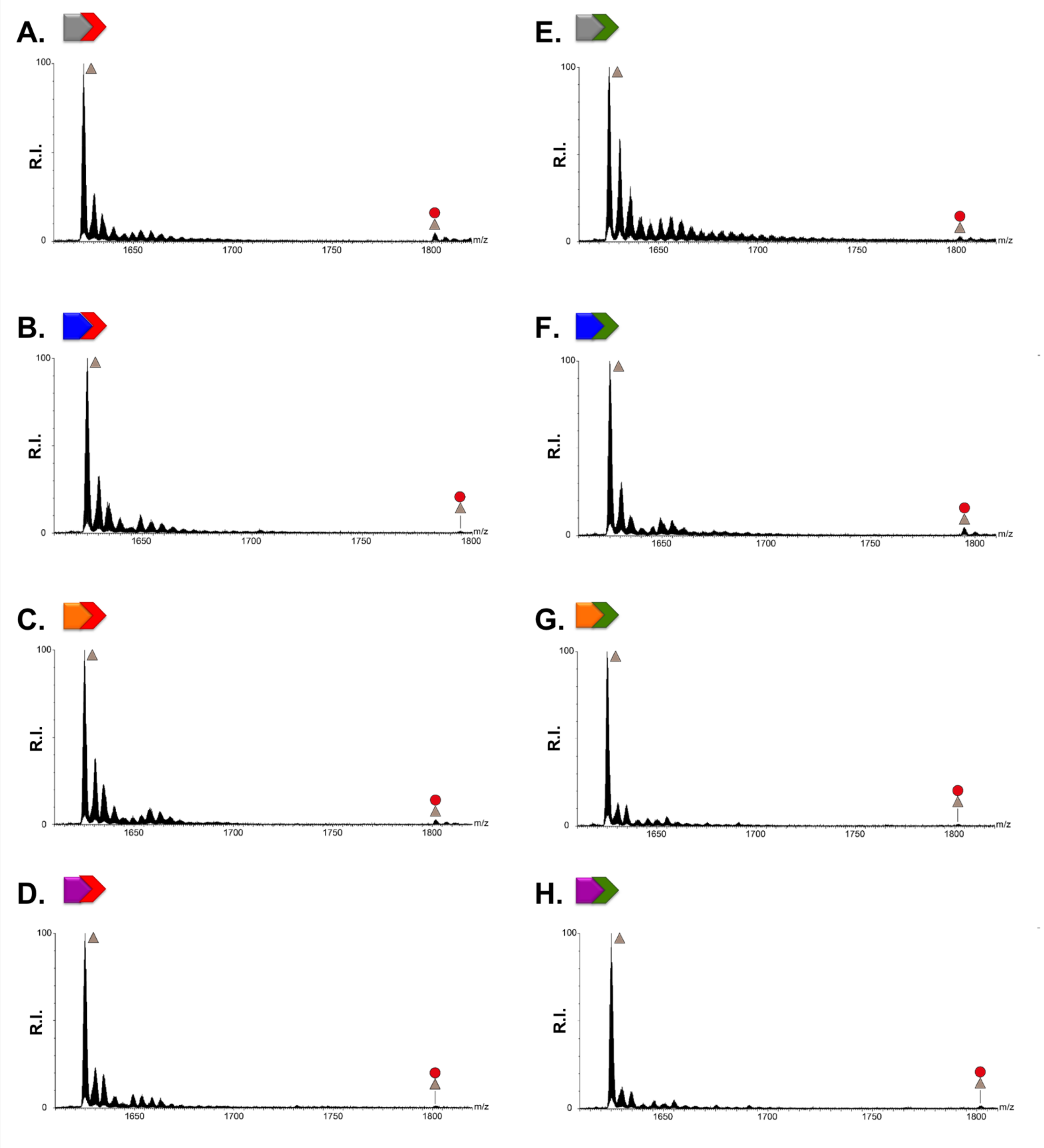

Figure S4. Representative ESI-MS spectra of samples obtained by mixing Tat with compounds of the L-Tyr and D-Tyr series. In the figure are reported the ESI-MS spectra acquired in positive ion mode of samples containing 10 µM of Tat (**A**), and obtained mixing 10 µM of Tat with 100 µM of **ß-Ala-Y** (**B**) in 150 mM ammonium acetate. In both spectra A and B, the peak detected at m/z 698.98 corresponds to the Tat protein. In spectrum B, compound **ß-Ala-Y** was identified at m/z 707.27. Lower intensity signals consist of typical sodium and ammonium adducts.

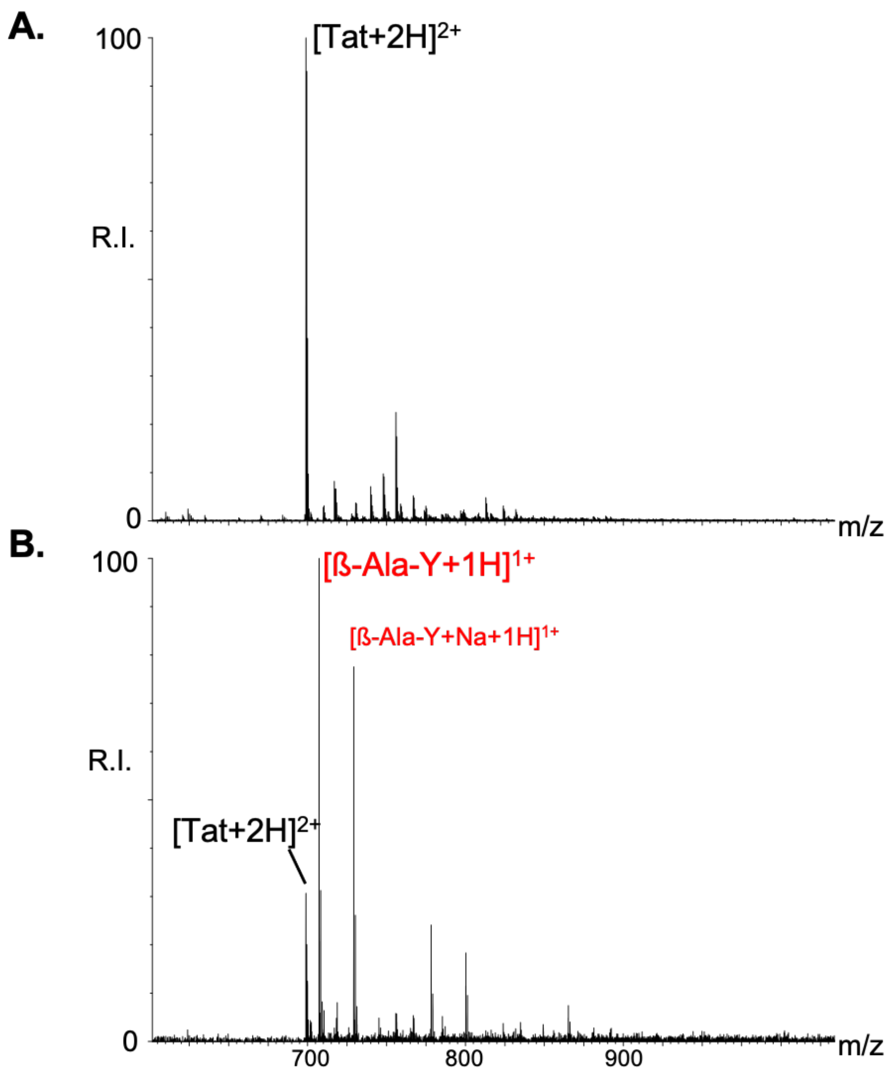

Table S4. Experimental and calculated monoisotopic masses (Da) of 2,6-AQ:Tat complexes. Expected masses of 1:1 complexes were calculated from the sequence of Tat protein (GRKKRRQRRR) and the elemental composition of each compound. No complexes were detected in the ESI-MS spectra.

Compound	1:1 AQ:Tat complex	
	Experimental Mass (Da)	Calculated Mass (Da)
**ß-Ala-Y**	Not detected	2102.19
**G-Y**	Not detected	2074.17
**A-Y**	Not detected	2102.19
**DA-Y**	Not detected	2102.19
**ß-Ala-DY**	Not detected	2102.19
**G-DY**	Not detected	2074.17
**A-DY**	Not detected	2102.19
**DA-DY**	Not detected	2102.19

Figure S5. ESI-MS spectra of samples obtained by adding NC to the preformed ligand•TAR complexes. The tested compounds are (**A**) **A-Y**, (**B**) **A-DY**, (**C**) **DA-Y**, (**D**) **DA-DY**. Lower intensity signals near free/bound species consist of typical sodium and ammonium adducts. Gray ▲ corresponds to NC protein; ■ to the TAR RNA substrate; and red ● to the ligand. For the sake of clarity only the 5+ and 6+ charge states of the binary (RNA-ligand) and ternary (RNA-ligand- protein) complexes are labeled in the spectra.

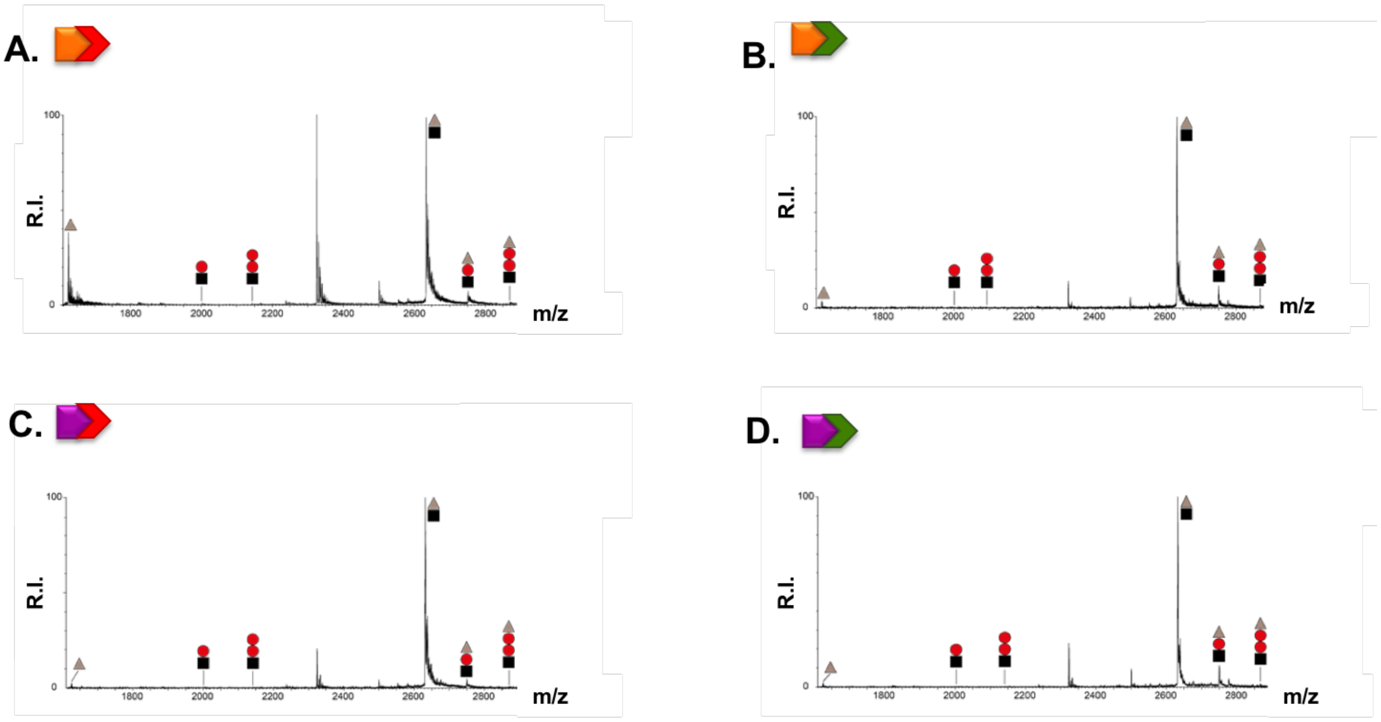

Figure S6. Parallel Artificial Membrane Permeation Assays (PAMPA). The graph reports the percentage of each compound detected in each compartment after 24 hours of incubation. In these assays, we included **ß-Ala-DK** and **ß-Ala-DOrn** as representative derivatives of the ß-Ala series of aliphatic charged compounds bearing D-Lysine and D-Ornithine as terminal residue, respectively.

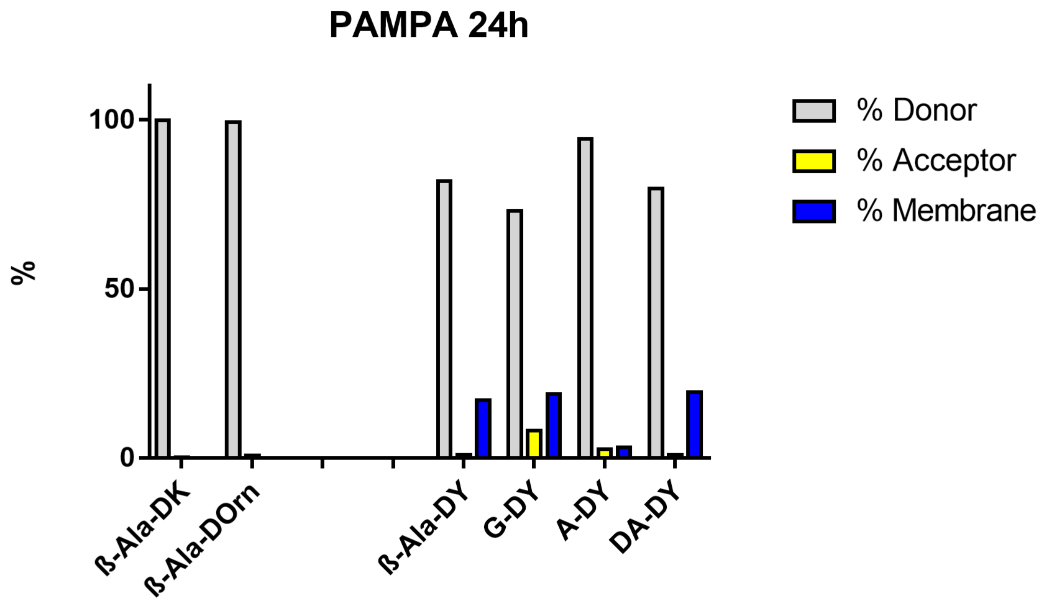

Supplement: Supplementary file 1 [file viruses-14-02133-s001.zip › viruses-1939750-supplementary.pdf]
